# Education Regarding and Adherence to Recommended Nutrition Guidelines among Dental Students

**DOI:** 10.3390/dj9080093

**Published:** 2021-08-09

**Authors:** Camille Frayna, Christoffer Devantier, Braden Harris, Karl Kingsley, Joshua M. Polanski

**Affiliations:** 1 Pediatric Dentistry Postgraduate Program, School of Dental Medicine, University of Nevada, 1700 W. Charleston Blvd., Las Vegas, NV 89106, USA; frayna@unlv.nevada.edu; 2 Department of Clinical Sciences, School of Dental Medicine, University of Nevada, 1700 W. Charleston Blvd., Las Vegas, NV 89106, USA; devantie@unlv.nevada.edu (C.D.); harrib11@unlv.nevada.edu (B.H.); 3 Department of Biomedical Sciences, School of Dental Medicine, University of Nevada, 1001 Shadow Lane, Las Vegas, NV 89106, USA; Joshua.Polanski@unlv.edu

**Keywords:** Dietary Guidelines for Americans (DGA), diet, dental student, education

## Abstract

The Dietary Guidelines for Americans (DGA) were developed to reduce or prevent many types of chronic illness, including cancer, heart disease and diabetes. Healthcare provider recommendations may be influenced by understanding of and adherence to the DGA, which may be incorporated into provider training, medical and dental clinical curricula—although few studies have evaluated adherence to the DGA among dental students. This approved retrospective study of voluntary student responses from a first-year dental school nutrition course included a short dietary and exercise survey administered as part of the DGA learning module. A total of N = 299 students completed the voluntary nutrition survey, yielding a response rate of 91.4%. Daily fruit and vegetable intake, dairy and whole grain servings among UNLV-SDM students were significantly lower than the DGA recommendations but higher than U.S. averages for 18–30-year-olds—although neither group met DGA recommendations. This study represents one of the first to evaluate the dietary intake of U.S. dental students for comparison with the DGA for positive health behaviors. These data demonstrate a lack of adherence to the DGA among highly educated dental students and the need for the curricular inclusion of diet and nutrition into the dental school curriculum.

## 1. Introduction

The Dietary Guidelines for Americans (DGA) were developed by the Department of Health and Human Services (HHS) and the United States Department of Agriculture (USDA) to improve or maintain the health of the United States (U.S.) population [1,2]. Many of the recommendations emphasize the epidemiological and clinical findings of health promotion associated with the consumption of nutrient-dense foods, including fruits and vegetables [3,4]. Other important DGA recommendations include limitations on saturated and trans fats, added sugars, and sodium—mostly associated with adverse health outcomes, morbidity and premature mortality [5,6,7].

Healthcare providers are encouraged to promote the DGA in patient consultations to limit or prevent many chronic illnesses, such as diabetes and heart disease [8,9]. Most of the evidence regarding DGA recommendations and implementations has traditionally involved primary care physicians, which have been seen as crucial in the efforts to reduce the burden of chronic diseases associated with poor dietary intake and behaviors [10,11,12]. However, new models of health information incorporate the use of alternative providers and educators to deliver DGA recommendations and dietary information, including teachers and other healthcare specialist providers [13,14].

Dentists are a specific subgroup of healthcare providers that have moderate to lengthy patient interactions which may benefit from these consultations, because the etiologies of many oral diseases are based in nutritional deficiencies, poor dietary intake and chronic illness, such as diabetes [15,16,17]. More specifically, pediatric dentists are seen as a key group of healthcare providers that can positively and significantly influence parental and pediatric patient dietary behaviors and specific policy recommendations have been made by the American Academy of Pediatric Dentistry to improve adherence to the DGA [18,19,20].

Some evidence now suggests that medical provider recommendations may be influenced by the understanding of and adherence to the DGA, which may be incorporated into medical education and clinical curricula [21,22,23]. In fact, the Academy of Nutrition and Dietetics has indicated interprofessional education in nutrition as an essential component of healthcare provider training and is indispensable for developing providers with the competence to implement this into clinical practice [24,25]. Although some studies have focused on the importance of including nutrition education among dental and dental hygiene students, few studies have evaluated adherence to the DGA among dental students [26,27,28]. 

Based upon this paucity of information in this area and the overall importance of this topic, the goal of this project was to evaluate the diet and nutrition of dental students compared with the USDA and DGA nutrition guidelines during the instruction of a dental school nutrition course.

## 2. Materials and Methods

### 2.1. Human Subjects

The protocol for this study titled “Retrospective analysis of health status of dental student population” was reviewed and approved by the Office for the Protection of Research Subjects (OPRS) and Institutional Review Board (IRB) of the University of Nevada, Las Vegas (UNLV), on 21 May 2020 [Protocol 1607120-2]. Informed consent was waived due to the exemption to human subject research under the Basic HHS Policy for Protection of Human Research Subjects, (46.101) Subpart A (b) regarding IRB exemption for research involving the use of education tests (cognitive, diagnostic, aptitude, achievement) in which the subjects cannot be identified directly or through identifiers.

Briefly, this study involved a retrospective review of voluntary student responses previously collected as part of the Clinical Nutrition course at UNLV-SDM for first-year dental students. All student responses were anonymous with no personal identifying information; only basic demographic information (age, sex, race/ethnicity) was collected with each set of dietary and exercise behaviors.

### 2.2. Diet and Exercise Survey

The voluntary student diet and exercise questionnaire consisted of N = 17 questions, which corresponded to the basic parameters of the current DGA recommendations. These included questions regarding fruit and vegetable intake, sugar-sweetened beverages, and the frequency and intensity of physical activity.

### 2.3. Statistical Analysis

Each of the diet and exercise survey responses were tabulated and basic descriptive statistics associated with these respondents were reported with summary statistics for the percentage of responses from each question. Differences between dental (DMD) student responses and the Dietary Guidelines for Americans (DGA) were assessed through chi-squared analysis using online software from GraphPad (San Diego, CA, USA).

## 3. Results

This NHANES-based survey was administered over multiple years to first-year dental students during a nutrition course, with a total of n = 327 students receiving the voluntary survey (Table 1). In brief, a total of n = 299 students participated and completed the survey, yielding an overall response rate of 91.4%. The percentage of males and females in the survey (42% and 58%, respectively) was not significantly different from the overall student population from which the samples were taken (41%, 59%, respectively), *p* = 0.8389. In addition, the percentage of students identifying as White/Caucasian/non-minority among the study sample was 40.5%, which was not significantly different from the overall student population (48%), *p* = 0.1612. Most of these minority students self-identified as Asian.

**Figure 1:**
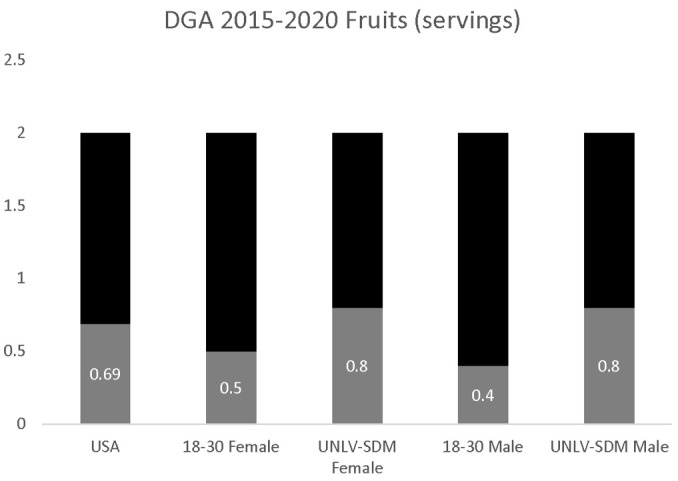
DGA fruit consumption analysis. Analysis of UNLV-SDM dental students’ fruit consumption among females and males, which was significantly higher than the U.S. population in general and young adults (18 to 30-year olds), more specifically, *p* = 0.031, but no significant differences were found between males and females, *p* = 0.899, and did not meet the DGA recommendations.

The estimate of overall fruit servings per day (0.69) was significantly lower than the DGA recommendations of 2.0. In addition, fruit consumption among 18–30-year-old females (0.5 servings) and males (0.4 servings) was also significantly lower than the DGA recommendations. Analysis of the data from UNLV-SDM dental students found no significant differences between males and females, *p* = 0.899. In addition, fruit consumption among females and males (0.8 servings per day) was significantly higher than the U.S. population in general and young adults (18–30), more specifically, *p* = 0.031 (Figure 1).

**Figure 2:**
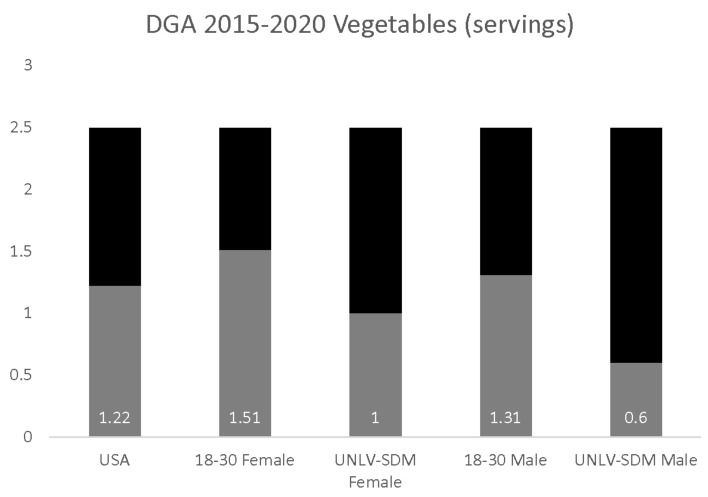
DGA vegetable consumption analysis. Analysis of UNLV-SDM dental students revealed significant differences, with consumption among females significantly higher than males, *p* = 0.022—although this was lower than the U.S. population in general and 18 to 30-year-olds, more specifically, *p* = 0.018, and did not meet the DGA recommendations.

The estimate of overall vegetable servings per day (1.22) was significantly lower than the DGA recommendations of 2.5. In addition, vegetable consumption among 18–30-year-old females (1.51 servings) and males (1.31 servings) was also significantly lower than the DGA recommendations (Figure 2). Analysis of the data from UNLV-SDM dental students found significant differences between males and females. Reported vegetable consumption among females (1.0 servings per day) was significantly higher than males (0.6 servings per day), *p* = 0.022—and lower than the U.S. population in general and young adults (18–30), more specifically, *p* = 0.018.

**Figure 3:**
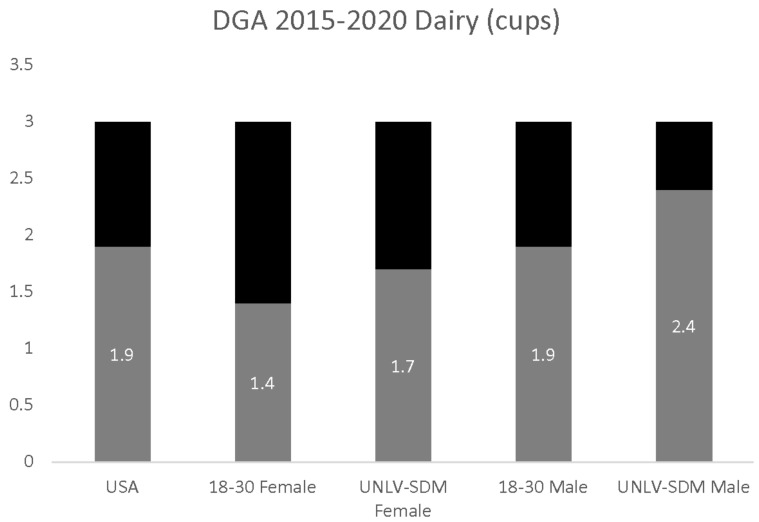
DGA dairy consumption analysis. UNLV-SDM dental student females consumed significantly less than males, *p* = 0.0014—although this was higher than observed among 18 to 30-year-olds, *p* = 0.0388, but still did not meet the DGA recommendations.

The estimate of overall dairy consumption (1.9 cups per day) was significantly lower than the DGA recommendation of 3.0 cups. In addition, dairy consumption among 18–30-year-old females (1.4 cups) and males (1.9 cups) was also significantly lower than the DGA recommendations (Figure 3). Analysis of the data from UNLV-SDM dental students also found significant differences between males and females, with reported dairy consumption among females (1.7 cups per day) significantly lower than males (2.4 cups per day), *p* = 0.0014—and different from the U.S. population in general and young adults (18–30), more specifically, *p* = 0.0388.

**Figure 4:**
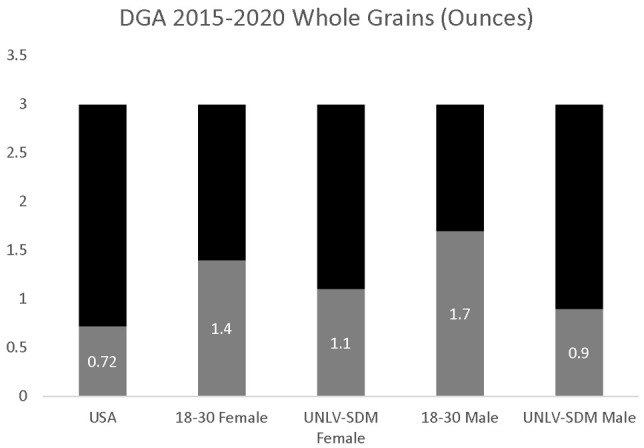
DGA whole grain consumption analysis. UNLV-SDM dental student females consumed nearly the same amount as males, *p* = 0.3687—although this was lower than observed among young adults between 18 and 30 years old, *p* = 0.0026, and did not meet the DGA recommendations.

Finally, the estimates of overall whole grain consumption (0.72 ounces per day) were significantly lower than the DGA recommendations of 3.0 ounces (Figure 4). In addition, whole grain consumption among 18–30-year-old females (1.4 ounces) and males (1.7 ounces) was also significantly lower than the DGA recommendations. Analysis of the data from UNLV-SDM dental students also found significant differences between males and females; the reported whole grain consumption among females (1.1 ounces per day) was not significantly higher than males (0.9 ounces per day), *p* = 0.3687, but was higher than the U.S. population in general and significantly lower than estimated among young adults (18–30 years old), more specifically, *p* = 0.0026.

## 4. Discussion

The primary objective of this study was to assess specific components of dietary consumption among dental students to compare with the DGA recommendations. Despite the highly educated nature of this study cohort, these results clearly demonstrated that dental students are no more likely to follow the DGA guidelines than other 18–30-year-olds in the United States. These data may also suggest that more in-depth integration of nutrition education, screening, counseling and referral may be needed to improve dental students’ knowledge, awareness and compliance with DGA recommendations and the subsequent incorporation into dental practice [29,30].

The results of this study may become increasingly important as other studies confirm the role of proper diets in maintaining oral health and the prevention of oral disease, such as the role of dental erosion and caries associated with the intake of sugar-sweetened beverages and other beverages with a strong acidic content [31,32]. In fact, many studies have specifically examined the role of diet, the maintenance of oral health, and the prevention of disease, although have increasingly focused on the consumption of extrinsic (added) dietary sugars and the relationship with coronal dental caries [33,34]. However, prominent calls for the inclusion of dietary advice into dental practice have not yet become fully integrated due to the lack of standardization in dental education regarding dietary consumption patterns, how these compare with DGA recommendations, and the influence of these behaviors on oral and systemic health [35].

This study may also be important to compare and contrast with the current status of dietary studies in dental education, which tend to focus more specifically on dietary knowledge, practices and screening, specific to caries development, progression and prevention—such as the frequency of consumption and total levels of dietary sugar intake [36,37]. Although an understanding of the relationship between sugar-sweetened beverages and sugar-rich diets with dental caries is an essential component of the dental curriculum, the understanding of proper nutrition and positive dietary practices, such as the consumption of fruits, vegetables, whole grain and dairy and their role in maintaining and preventing oral diseases is also critically important, such as the role of dietary vitamin C and B12 in preventing gingival bleeding and periodontal disorders by maintaining collagen and extracellular matrix integrity [38,39,40]. Indeed, many other oral conditions, including periodontal disease, disorders of the oral mucosa and infectious agents, are also mediated by dietary behaviors and could be improved with dental provider recommendations and targeted dietary counseling and education [41].

Despite the significance of these results, there are some limitations associated with this study that should be addressed and considered. First, this was a retrospective, cross-sectional study, and therefore did not address any potential changes in dietary behavior following the completion of the nutrition course or DGA modules [42,43]. No data were collected from alumni or graduates, which could potentially demonstrate that this curricular component may positively influence dental student dietary choices and behaviors. In addition, this study did not fully assess or estimate the dietary consumption and intake of other items associated with poor health outcomes and negative health behaviors, such as added salt, sugar and fat, or low-nutrient-dense snack foods containing these ingredients [44]. Incorporation of these items into a more comprehensive dietary assessment may facilitate integration of these data into additional areas of the dental school curriculum, such as dental public health, caries and periodontology concepts.

However, many new innovations have been developed to teach and integrate nutrition education in medical and dental curricula in recent years [45]. For example, a new interactive teaching and learning platform, “Health Meets Food”, features specifically designed online interactive course modules, continuing education credits and online videos to complement and enhance applied nutrition education in clinical training programs, including medicine and dentistry [46,47]. It has been demonstrated that these interactive courses and education modules may improve and achieve dietary sodium recommendations among cardiovascular patients, as well as reducing heart-failure-associated readmissions and improving HbA1c, blood pressure and cholesterol levels for patients with adult onset or Type II diabetes [48,49,50].

These innovations rely on the ability to identify dietary and culinary deficiencies among graduate and professional students during their clinical education and curriculum in order to appropriately integrate their training towards healthy cooking and eating—an important first step before these recommendations can be taught to their patients [51,52]. The ability to then supplement and apply this training towards specific health conditions, such as nutrition for cancer prevention and supplemental treatment, then becomes feasible [53]. These (and other) studies have clearly demonstrated the potential to significantly modify not only the attitudes, behaviors, and knowledge of graduate and professional healthcare students during their training, but also to influence the integration of these attitudes, knowledge and behavior towards their clinical training and recommendations during patient care [54,55,56,57].

## 5. Conclusions

This study may be among the first to evaluate the dietary intake of U.S. dental students for comparison with the DGA for positive health behaviors, including the consumption of fruits, vegetables, dairy and whole grains. The results of this study highlight the need for the curricular integration of diet and nutrition into dental and healthcare curricula due to the low levels of adherence among highly educated doctoral-level students in training. Future studies may be needed to evaluate the impact of these educational initiatives and measure any changes in dietary behaviors following the completion of these modules and courses.

## Figures and Tables

**Table 1:** Demographic characteristics of dental student participants.

	Respondents (n = 299)	Total Student Population	Statistical Analysis
Females	n = 126/299 (42.1%)	41%	χ^2^ = 0.041, d.f. = 1
Males	n = 173/299 (57.9%)	59%	*p* = 0.8389
Caucasian/White	n = 121/299 (40.5%)	48%	χ^2^ = 1.963, d.f. = 1
Minority/non-White	n = 178/299 (59.5%)	52%	*p* = 0.1612
Asian	n = 123/299 (41.1%)	40%	
Hispanic/Black/Other	n = 55/299 (18.4%)	12%	

## Data Availability

The data presented in this study are available on request from the corresponding author. The data are not publicly available due to the study protocol data protection parameters requested by the IRB and OPRS for the initial study approval.
